# Lv4, an activity that restricts nuclear entry of SIV_MAC_/SIV_SM_ in human blood cells

**DOI:** 10.1186/1742-4690-10-S1-O28

**Published:** 2013-09-19

**Authors:** Massimo Pizzato, Martha Neagu, Thomas Pertel, Claudia Firrito, Serena Ziglio, Madeleine Zufferey, Lionel Berthoux, Jeremy Luban

**Affiliations:** 1 Microbiology and Molecular Medicine, University of Geneva, Geneva, Switzerland; 2 Center for Integrative Biology, University of Trento, Trento, Italy; 3 Laboratory of Retrovirology, University of Quebec, Trois-Rivières, Canada; 4 Program in Molecular Medicine, University of Massachusetts Medical School, Worcester, USA

## 

SIV_SM_ is a lentivirus endemic to the West African sooty mangabey (*Cercocebus atys*)*.* HIV-2 and SIV_MAC_ are zoonoses that resulted from SIV_SM_ transmission to humans and Asian rhesus macaques (*Macaca mulatto*), respectively. Human leukemia cell lines, human peripheral blood mononuclear cells and CD4^+^ T cells, were 4 to 50-fold less permissive for SIV_MAC_ and SIV_SM_ than for HIV-1. In contrast, SIV_MAC_ transduction of human adherent cell lines was equivalent to that of HIV-1. Consistent with adaptation to human cells, HIV-2 was not restricted as potently as was SIV_MAC_. SIV_MAC_ transduction of human blood cells was rescued up to the level of HIV-1 by As_2_O_3_, a compound that increases the infectivity of viruses in the context of TRIM5-mediated restriction. Nonetheless, efficient knockdown of TRIM5 or cyclophilin A, a cytoplasmic factor that sometimes regulates TRIM5 restriction activity, did not rescue SIV_MAC_ tranduction of these cells. Substitution of HIV-1 CA with the CA from SIV_MAC_ rendered HIV-1 poorly infectious for Jurkat T cells. The block occurred after completion of reverse transcription and the formation of 2-LTR circles, but before establishment of the provirus. Heterokaryons resulting from fusion of permissive with restrictive cells exhibited the restrictive phenotype, indicating that SIV transduction of human blood cells is inefficient due to a dominant-acting restriction factor. These results demonstrate that the nucleus of human blood cells possesses a TRIM5-like restriction factor specific for the SIV_MAC_/SIV_SM_ capsid and that, by extension, cross-species transmission of *SIV_SM_* to human cells necessitated adaptation of HIV-2 to this restriction factor.

